# Data on the adoption of pesticide-free wheat production in Switzerland

**DOI:** 10.1016/j.dib.2022.107867

**Published:** 2022-01-23

**Authors:** Niklas Möhring, Robert Finger

**Affiliations:** a Agricultural Economics and Policy Group, ETH Zürich, Sonneggstrasse 33, 8006 Zürich, Switzerland; b Centre d'Etudes Biologiques de Chizé, UMR 7372, CNRS and La Rochelle Université, Beauvoir-sur-Niort, France

**Keywords:** Pesticide, Pesticide-free, Sustainable agriculture, Adoption, Wheat, Public-private, Sustainability standard, Switzerland

## Abstract

The reduction of environmental and health risks from pesticide use is on top of the agenda of the food-value chain actors. The establishment of pesticide-free production systems could be a cornerstone for the sustainable intensification of agriculture. In Switzerland, a pesticide-free but non-organic wheat production standard is currently being introduced. We present survey data of 1105 IP-SUISSE producers on adoption, future adoption, expectations and perceptions of the program, structural farm, and farmers' characteristics, and producers' risk preferences, farming objectives, attitudes and goals, self-efficacy, and locus of control. The data was collected to identify adoption determinants, barriers, and incentives for pesticide-free wheat production. The data is combined with publicly available data on soil properties, climate, weed pressure, and spread of herbicide resistance.

## Specifications Table

**Table:** 

Subject	Agricultural economics; Agriculture
Specific subject area	Adoption of sustainable (pesticide-free) production, preferences, expectations, non-cognitive skills, structural farm and environmental data.
Type of data	CSV file
How data were acquired	An online survey combined with structural farm-level and environmental data. Limesurvey. R.
Data format	Raw and partly filtered (for reasons of confidentiality).
Parameters for data collection	IP-SUISSE wheat producers in Switzerland.
Description of data collection	The online questionnaire was distributed via Limesurvey to the entire population of IP-SUISSE wheat producers in Switzerland (4749). Participation was incentivized. In total, 1105 respondents completed the survey. The data was anonymized.
Data source location	We collect data from farmers that are situated all over Switzerland (see [1] and Fig. 1 for an overview map of locations).
Data accessibility	Repository name: ETH Zürich Research Collection Data identification number (permanent identifier, i.e. DOI number): 10.3929/ethz-b-000450297 Direct link to the dataset: https://doi.org/10.3929/ethz-b-000450297

## Value of the Data

• Pesticide-free production systems can substantially contribute to pesticide risk reduction - a topic of societal and policy relevance. We here provide data on the adoption of pesticide-free wheat production in Switzerland, which gives a comprehensive overview of potential adoption determinants and barriers for pesticide-free production.
• Researchers, policymakers and food-value chain actors aiming to understand the determinants and barriers of pesticide-free production can profit from this dataset.
• This data can be used in the future to provide further inisghts on socio-economic, environmental and farmers intrinsic drivers for the adoption of sustainable production practices in agriculture

## 1 Data Description

We collected survey data from IP-SUISSE wheat producers in Switzerland. More specifically, we focused on the adoption of a pesticide-free but non-organic wheat production standard in the growing season 2019/20. The standard was first introduced as a pilot in the growing season 2018/19 and was opened up for public participation in the growing season 2019/20. The survey was designed based on a conceptual adoption model discussed with farmers, extension service experts, and farm advisors. The initial survey was pre-tested with ten producers. It was sent out to the entire population of IP-SUISSE wheat producers (4749). IP-SUISSE producers already produce wheat under an extensive production scheme of the producer organization IP-SUISSE, which prohibits the use of fungicides and insecticides in wheat production. The pesticide-free production scheme goes one step further and prohibits the use of all synthetic pesticide in wheat production. The survey data comprise 1105 complete responses (response rate of 23.3%) of producers. Survey data include information on adoption as well as potential adoption in the future.

**Fig. 1:**
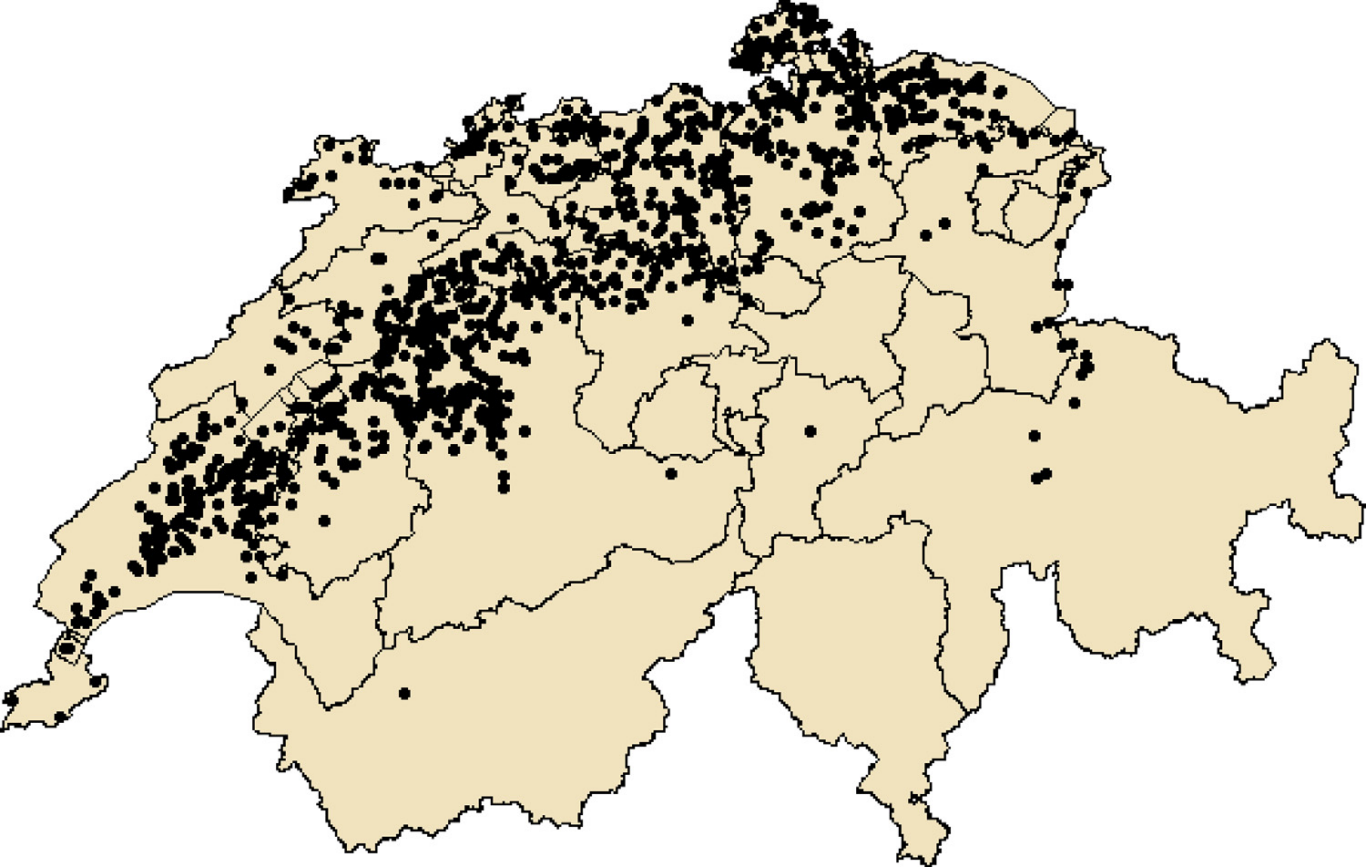
Map of survey participants in Switzerland.

Moreover, expectations and perceptions of the program and farm and producers' characteristics were surveyed. We further collected producers' risk preferences, farming objectives, attitudes, goals, and non-cognitive skills such as self-efficacy and locus of control. Survey respondents were distributed all over Swiss wheat production regions (see Fig. 1). We combined the survey data with publically available data on i) soil properties from the Swiss Federal Office for Agriculture [2], ii) climate from MeteoSuisse [3,4], iii) weed pressure from Info Flora [5] and iv) spread of herbicide resistances from Agroscope [6], matched by farm location, respectively.

All survey data and metadata are available from the ETH Zürich Research Collection: https://doi.org/10.3929/ethz-b-000450297, including:

- A readme file indicating all data files in the repository entry in text format.
- An overview file, shortly describing variables of the survey data in PDF format.
- The here described survey data with original variable names in csv format.
- The here described survey data with English variable names in csv format.
- The original questinnaires used (in German and French) in PDF format.
- The original questionnaires translated in English in PDF format.

We combined survey data with external data sources based on farm location. See Table 1 for a description of all data sources used. For a complete description of all variables in the dataset, see the codebook, which is available together with the dataset under the above link.

**Table 1:** Overview of used data sources.

Data	Short description	Reference
Survey	See section 2 below.	Described in this article.
Soil properties	Soil suitability for wheat production.	(Swiss Federal Office for Agriculture, 2009) [2]
Climate	Average yearly temperature and precipitation in the wheat-growing season.	(Frei, 2014; Frei et al., 2006) [3,4]
Weed pressure	Regional weed abundance, according to Info Flora.	(Info Flora, 2019) [5]
Herbicide resistances	The spread of herbicide resistances per municipality.	(Tschuy and Wirth, 2015) [6]

## 2 Experimental Design, Materials and Methods

The online survey link was distributed in December 2019 via E-Mail to all IP-SUISSE wheat producers in Switzerland (4749). At this point, producers had already decided to participate in the program for the growing season 2019/20. In the E-Mail, producers were encouraged to participate by IP-SUISSE. Furthermore, participation was incentivized with a lottery of 20 times 50 CHF supermarket vouchers among those participants completing the survey. The E-Mail highlighted that participation, both of producers, with positive and negative attitudes towards the program, is welcomed. We used Limesurvey to conduct the study and collect the survey data. Extension service experts and experts from IP-SUISSE reviewed the survey. It was then pre-tested with 10 IP-SUISSE wheat producers. The survey was online for five weeks, and two reminders were sent out after 2 and 4 weeks, respectively. In the survey, producers could choose between a German and a French version of the survey. In the introduction to the survey, its purpose was clearly explained again. Participants were reassured at the beginning of each of the three survey sections that their answers will be treated completely anonymous.

We merged the survey data with publically available data on soil properties, climate, weed pressure, and spread of herbicide resistances from Agroscope by farm location (municipality and ZIP code level), respectively.

The survey contained 22 questions, and answering the questionnaire took producers a median time of 17.9 minutes. The structure of the survey was as follows:

1. Participation and future participation in the IP-SUISSE program for pesticide-free wheat production and expectations towards the program's effects.
2. Personal characteristics of the producer and characteristics of the farm
3. Preferences and perceptions

### 2.1 Participation in the IP-SUISSE program for pesticide-free wheat production

In this part of the questionnaire, producers were first asked to state if they had participated in the pesticide-free wheat production program in 2018/19, if they were inscribed for 2019/20 or if they intend to participate in the future. The following questions were reframed depending on their answers, referring either to experiences or plans when adopting the program. Furthermore, farmers were asked about participation in direct payment programs for soil conservation (federal) and pesticide use reduction (both federal and cantonal). We then asked them to evaluate decisive factors to participate/not participate in the program on a scale from 1-5, to state their experience with pesticide-free wheat production, their (intended) strategy for weed management under adoption, and availability of machinery to deploy these strategies. In the next questions of this part of the survey, we asked producers to state their perceived risks for machinery investment on a scale from 1-5, to evaluate the main factors for these risks, again on a scale from 1-5, and to indicate their main information sources for weed management. We concluded this section with questions about the producers' expectations concerning potential yield reduction and production risk increases under program adoption.

### 2.2 Personal characteristics of the producer and characteristics of the farm

This section of the survey asked producers to indicate on-farm workforce, which part of their wheat production surface was leased, income shares in arable, animal, off-farm, and other production, education, age, and farm succession.

### 2.3 Preferences and perceptions

In the third section of the survey, producers were first asked to self-elicit their risk preferences on a scale from 0-10 in the domains of plant protection, agricultural production, marketing, and general decisions on the farm. Afterward, we asked them to evaluate their perception of the program's effects on the environment and human health, farming objectives, environmental preferences in farming, and openness for innovations and consultations with neighbors on a scale from 0-5. We then asked two questions, each concerning the producers' self-efficacy and locus of control, framed on wheat production and plant protection, respectively. Finally, we gave participants the possibility to leave comments.

## Ethics Statement

All methods were carried out in accordance with guidelines and regulations at ETH Zurich, and informed consent was obtained from all subjects prior to entering the survey. The project and study were approved by the office of research of ETH Zurich, and the survey was approved by the board of IP Suisse, the farmers’ association representing participants of the study.

## CRediT Author Statement

**Niklas Möhring:** Conceptualization, Methodology, Validation, Data Collection, Data Curation, Writing – Original draft and Review &Editing, Visualization, Funding acquisition; **Robert Finger:** Conceptualization, Methodology, Data curation, Writing – Original draft, Funding acquisition.

## Declaration of Competing Interest

The authors declare that they have no known competing financial interests or personal relationships that could have influenced the work reported in this paper.
